# Characterization of the erythrocyte GTPase Rac1 in relation to *Plasmodium falciparum* invasion

**DOI:** 10.1038/s41598-020-79052-0

**Published:** 2020-12-16

**Authors:** Silvio Paone, Sarah D’Alessandro, Silvia Parapini, Francesco Celani, Valentina Tirelli, Manoochehr Pourshaban, Anna Olivieri

**Affiliations:** 1 Dipartimento di Malattie Infettive, Istituto Superiore di Sanità, Rome, Italy; 2 Dipartimento di Sanità Pubblica e Malattie Infettive, Sapienza University of Rome, Rome, Italy; 3 Dipartimento di Scienze Biomediche, Chirurgiche e Odontoiatriche, University of Milan, Milan, Italy; 4 Dipartimento di Scienze Biomediche Per La Salute, University of Milan, Milan, Italy

**Keywords:** Parasite biology, Target identification

## Abstract

Malaria is still a devastating disease with 228 million cases globally and 405,000 lethal outcomes in 2018, mainly in children under five years of age. The threat of emerging malaria strains resistant to currently available drugs has made the search for novel drug targets compelling. The process by which *Plasmodium falciparum* parasites invade the host cell has been widely studied, but only a few erythrocyte proteins involved in this process have been identified so far. The erythrocyte protein Rac1 is a GTPase that plays an important role in host cell invasion by many intracellular pathogens. Here we show that Rac1 is recruited in proximity to the site of parasite entry during *P. falciparum* invasion process and that subsequently localizes to the parasitophorous vacuole membrane. We also suggest that this GTPase may be involved in erythrocyte invasion by *P. falciparum*, by testing the effect of specific Rac1 inhibitory compounds. Finally, we suggest a secondary role of the erythrocyte GTPase also in parasite intracellular development. We here characterize a new erythrocyte protein potentially involved in *P. falciparum* invasion of the host cell and propose the human GTPase Rac1 as a novel and promising antimalarial drug target.

## Introduction

Malaria is a devastating disease with 228 million cases globally and 405,000 lethal outcomes in 2018, mainly in children under five years of age. Around 90% of malaria cases and 94% of fatal outcomes occur in sub-Saharan Africa, where *Plasmodium falciparum*, the most lethal among *Plasmodium* species is predominant^1^. Parasite strains resistant to currently available antimalarial drugs periodically emerge. Since 2014, drug-resistance has been reported for all the existing classes of antimalarial compounds and the threat of emerging malaria strains has made the search for novel drug targets compelling^2–5^.

*Plasmodium falciparum* has a complex life cycle, undergoing its sexual reproduction in *Anopheles* mosquitoes and asexual reproduction in the human host. After a first reproductive cycle in the liver, the merozoites, free invasive forms of the parasite, penetrate inside human erythrocytes, where they mature and reproduce by schizogony. Mature schizonts then break the host cell and release new invasive merozoites in the bloodstream. Invasion of red blood cells (RBCs) by *Plasmodium* merozoites is a multi-step process, involving four different phases. After a first adhesion of the merozoite to the cell membrane (i), it re-orientates apposing its apical end to the erythrocyte membrane (ii). Then the parasite releases the content of its apical organelles and forms a tight junction (called “moving junction”) with the RBC membrane, sealing the plasma membrane of the two cells (iii). Finally the parasite enters into the host erythrocyte, forming the parasitophorous vacuole membrane (PVM), derived from the host plasma membrane (iv)^6^. An important consideration in terms of drug-development is that erythrocyte invasion requires a series of coordinated and often irreversible events to occur in sequence, with even small perturbations of this complex process likely to limit parasite survival in vivo. Despite *Plasmodium* invasion of the host cell has been widely studied, only a few erythrocyte proteins involved in this process have been identified so far^7–10^.

In the attempt to identify novel erythrocyte proteins involved in the malaria parasite invasion process, we focused our attention on the human GTPase Rac1, a member of the Rho GTPase family. These proteins are found in most eukaryotes, but are absent in *P. falciparum*^11^. Rac1 rules several molecular processes involving actin regulation, such as lamellipodia formation, membrane ruffling and cell division^12^ and, more importantly for this work, is involved in invasion of the host cell by many intracellular pathogens^13^. Like all the GTPases, Rac1 acts as a switch by cycling between an active (GTP-bound) and an inactive (GDP-bound) conformation, in response to chemical or physical stimuli. Its conformation is regulated by three classes of regulatory proteins: i) Guanine nucleotide Exchange Factors (GEFs), which promote exchange of GTP for GDP, thus activating Rac1 signaling; ii) GTPase-activating proteins (GAPs), which enhance the GTP-hydrolysis activity, shortening signal duration; and iii) guanine-nucleotide-dissociation inhibitors (GDIs), which maintain the GTPase in an off-state, preventing GDP dissociation and inactivating Rac1 signaling^14^.

In the erythropoietic line, Rac1 is involved in the proliferation and differentiation of erythroid precursors^15^ and in the formation of the contractile actin ring during erythroblast enucleation^16^. Rac1 conditional KO in Rac2 KO mice for the erythropoietic line led to erythrocytes with a disorganized membrane skeleton^17^. In mature erythrocytes, the role of Rac1 has not been elucidated. The GTPase could either have specific functions or just be residual.

Many intracellular bacteria, such as *Campylobacter jejuni*^18^, *Neisseria gonorrhoeae*^19^, *Staphylococcus aureus*^20^, *Listeria monocytogenes*^21^, *Shighella* species^22^, *Salmonella typhimurium*^23^ and others, activate Rac1 to invade the host cell, either leading to the formation of actin-rich membrane protrusions that facilitate pathogen entry, or to a localized disruption of the actin cytoskeleton that allows pathogen penetration into the host cell. Bacteria can modulate Rac1 activity either by injecting virulence effectors directly into the host cell through needle-like appendages^24^ or by secreting soluble virulence factors, that bind to the host cell surface and are then internalized by endocytosis. These factors can act both on proximal and distal host cells and are delivered to target cells by outer membrane vesicles produced by the bacteria^25,26^. Rac1 activation state can be modulated by the pathogen essentially in three ways: (i) by secreting factors that mimic Rac1 protein modulators, like GEFs, GAPs and GDIs^27^, (ii) by generating post translational modifications that act on Rac1 activation state or on its membrane localization^28^, or (iii) by regulating Rac1 degradation by the ubiquitin–proteasome system^29^.

Rac1 plays an important role also in infection by intracellular parasites such as *Trypanosoma cruzi*^30^, *Leishmania donovani*^31^ and interestingly also *Toxoplasma gondii*^32^, which belongs to the same phylum as the malaria parasite *Plasmodium falciparum*. In *T. gondii* the host GTPase is activated by parasite infection and is recruited to the PVM in its active form. Down-regulation of Rac1 in the host cell leads to a significant reduction in parasite invasion rates, demonstrating that Rac1 is necessary for the invasion of the host cell by *T. gondii*^32^.

Because of its role in the spread of many tumors, Rac1 has been extensively studied and several cell permeable Rac1 inhibitors have already been developed and are commercially available^33–36^. It has also been shown that treatment with Rac1-specific inhibitors reduces invasion rates of several intracellular bacteria^23,37–40^.

Here we suggest that Rac1 may play a role in erythrocyte invasion by *P. falciparum* and subsequent intracellular parasite growth and propose the human GTPase as a novel and promising antimalarial drug target.

## Results

### Rac1 is the only GTPase of the Rac family expressed in mature erythrocytes

The Rac GTPase family includes four members: Rac1, Rac2, Rac3 (sharing 88% of sequence identity) and RhoG, the latter being more divergent from the others (72% sequence identity) and consequently less prone to share redundant functions with the other three or to be recognized by antibodies and inhibitors specific for the other Rac GTPases^41^. Rac3 is known to be specifically expressed in the nervous system^42^ and animals knockout for this GTPase show no obvious erythropoietic phenotypes^43,44^. Rac1 and Rac2 instead, were shown to be expressed in the erythropoietic line^45^, but according to published data, only Rac1 has been identified by proteomic analysis in mature human erythrocytes (http://rbcc.hegelab.org).

**Figure 1:**
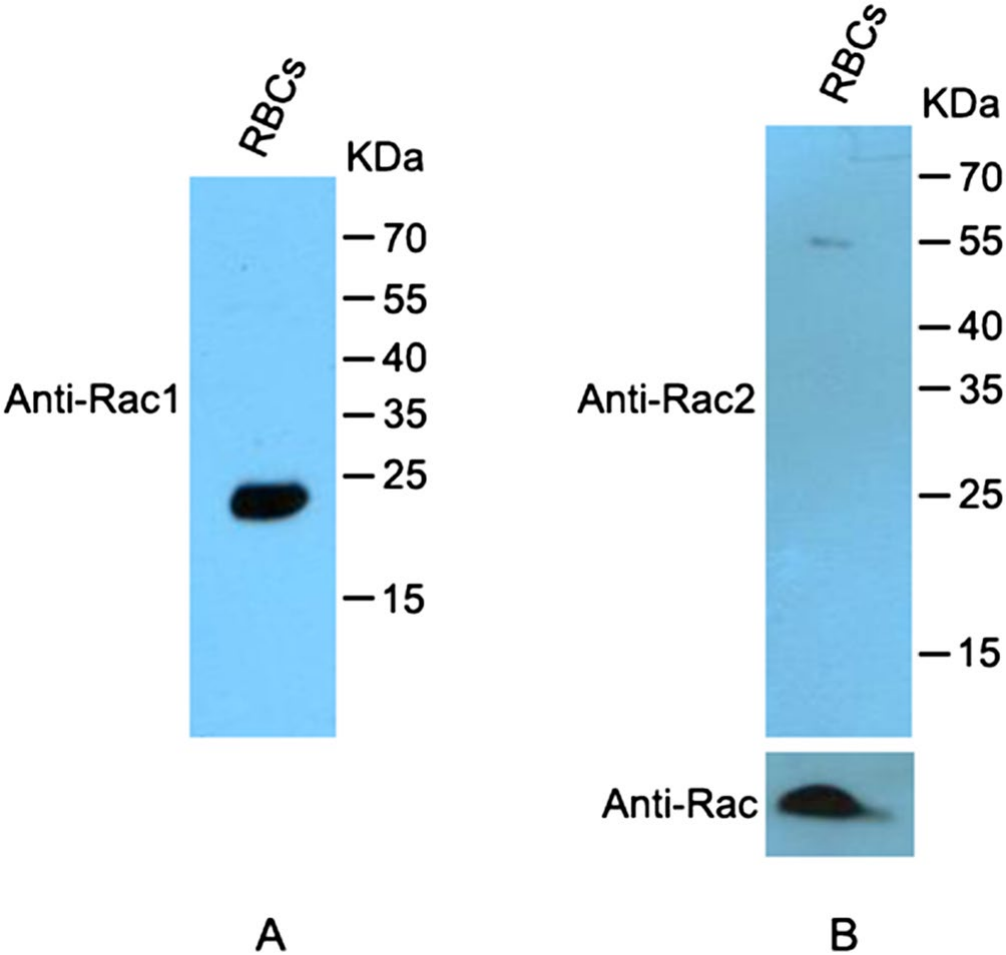
Rac expression in human erythrocytes. (**A**) Proteins extracted from of 5 × 10^7^ erythrocyte membranes were hybridized with a monoclonal antibody specific for Rac1 (R1-ab#2). The signal was detected at about 21 kDa, as expected. (**B**) In a different gel, same amounts of erythrocyte membranes were probed with the monoclonal anti-Rac2 specific antibody; no signal was detected. The same filter was then probed with an anti-Rac antibody (recognizing Rac1-3 proteins) (R1-ab#1), used as a loading control (bottom). The full lanes are shown in Fig. S10.

In order to confirm this, we purchased antibodies against Rac1 and Rac2, claimed by the manufacturer to be highly specific. All the anti-Rac1 antibodies used in this work are listed in Table S1. We first confirmed antibody specificity, by testing the anti-Rac1 and the anti-Rac2 antibodies on commercial GST-tagged Rac1 and histidine-tagged Rac2 proteins. Both antibodies only recognised the Rac protein they were raised against and did not give any signal on the other Rac protein (Fig. S1). Once demonstrated the antibody selectivity, we investigated Rac1 and Rac2 expression in mature human erythrocytes. Two samples of proteins extracted from erythrocyte membranes were analysed by western blot, either using the anti-Rac1 antibody or the anti-Rac2 antibody. While a significant signal was detected with the anti-Rac1 antibody, no signal was detected with the anti-Rac2 antibody on same amounts of membranes (Fig. 1A,B). This result confirms that Rac1 is the only detectable Rac GTPase in human mature erythrocytes, as reported by previous proteomic analyses (http://rbcc.hegelab.org).

### Rac1 subcellular localization and activation state

With the aim of investigating Rac1 subcellular localization in *P. falciparum* infected and non-infected erythrocytes, we performed immuno-fluorescence assays (IFAs) with commercial antibodies against Rac1. As a first step, we confirmed the absence of possible cross-reactions with parasite proteins, by testing anti-Rac1 antibodies in western blot against erythrocyte membranes and saponin-treated mixed asexual parasite stages, a treatment used for eliminating uninfected erythrocytes^46^ (Fig. S2). We also performed IFAs of mixed asexual parasites, to confirm that all the anti-Rac1 antibodies co-localized with each other (Fig. S3).

**Figure 2:**
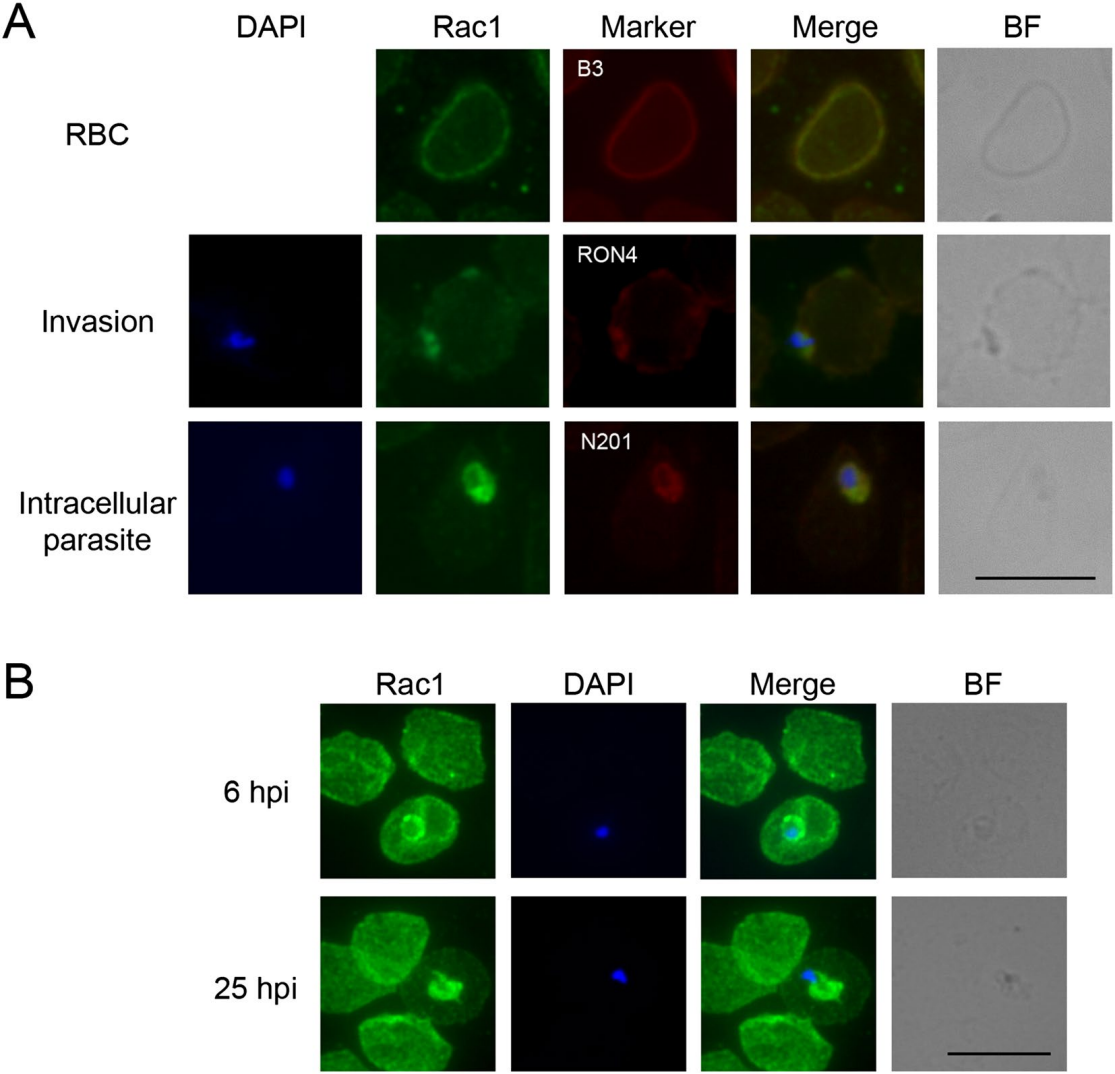
Rac1 subcellular localization. (**A**) IFAs of infected and non-infected human erythrocytes. Anti-Rac1 polyclonal antibody (ab#4) was used together with a primary antibody against Band3 (B3), as a marker of erythrocyte membrane, anti-RON4 antibody as a marker of the moving junction and anti-N201 as a marker of the PVM. RBC: non-infected erythrocyte. Invasion: parasite invading an erythrocyte. The nucleus has the typical bilobed shape. Intracellular parasite: parasite inside the parasitophorous vacuole. BF: Bright field. Nuclei are stained with DAPI. Different exposure times were used in each image. Scale bar: 10 µm. (**B**) IFA of synchronous parasites at 6 hpi and 25 hpi stained with the monoclonal anti-Rac1 R1-ab#3. BF: Bright field. Nuclei are stained with DAPI. Scale bar: 10 µm.

We then tested uninfected erythrocytes with two commercial polyclonal antibodies against Rac1 and an antibody against the integral membrane protein Band3. This showed that Rac1 in erythrocytes co-localizes with Band3 and suggests a membrane-associated localization, even though we cannot exclude that some of the protein may be cytoplasmic (Fig. 2A, S4 and S5).

To investigate Rac1 localization during *P. falciparum* invasion process, we produced parasite cultures enriched in invasive stages, the merozoites, by treating schizonts-infected erythrocytes with E64, a compound that reversibly inhibits merozoite egress^47^. When infected erythrocytes were washed and put back in culture medium, merozoites were released within minutes. We collected samples 5 min after cell washing and analysed this merozoite-enriched culture by IFA with a polyclonal anti-Rac1 and an antibody against the Rhoptry Neck Protein 4 (RON4), a moving junction marker^48^. These experiments showed that Rac1 localizes in proximity to RON4 (Pearson’s correlation coefficient = 0.89) (Fig. 2A), while it only partially colocalizes with the surface marker Merozoite Surface Protein 1 (MSP1)^49^ (Fig. S6), suggesting that the GTPase is recruited to the site of parasite entry upon invasion. More images are shown in Fig. S7A. The same sample was also hybridized with both the polyclonal anti-Rac1 and a monoclonal antibody that specifically recognizes the active form of Rac1 (anti-Rac1/GTP). This showed that part of the GTPase localizing to the invasion site is in the active conformation (Fig. S7B), even though we could not determine the relative amount, due to the high variability of the fluorescence signal around the invasion site.

To study Rac1 localization in the intraerythrocytic stages, we performed co-localization experiments with the PVM marker N201^50^, showing that the two proteins co-localize (Pearson’s correlation coefficient = 0.88) and that the GTPase is recruited to the PVM in infected erythrocytes (Fig. 2A). We also performed IFAs on synchronous stages. To obtain tightly synchronous parasite stages, purified schizonts were allowed to invade erythrocytes for 1 h and then those that did not release their daughter cells were killed by 5% sorbitol treatment, thus obtaining parasites born within a time window of 1 h. Blood smears were taken at 1 h post invasion (hpi), 6 hpi, 25 hpi and 42 hpi and analysed by IFA with a monoclonal anti-Rac1 antibody (Fig. S8). These experiments showed that at 6 hpi signal intensity on erythrocyte membrane and PVM is comparable, while at 25 hpi, when parasites progress to the trophozoite stage, the GTPase is almost completely depleted from the erythrocyte surface and relocated to the PVM (Fig. 2B and S8). These experiments showed that the GTPase is further recruited to the PVM during intraerythrocytic growth and suggest a possible role of the protein also in this phase of the life cycle. We could not determine precisely Rac1 localization in the schizont stage, because the signal is widespread (Fig. S8). The fluorescence signal becomes weaker and peripheral in the mature schizont (Fig. S9A). Merozoites show a very weak Rac1 signal, diffused on parasite surface, possibly due to residual Rac1 on membranes recycled by the schizont to generate daughter cells (Fig. S9B).

**Figure 3:**
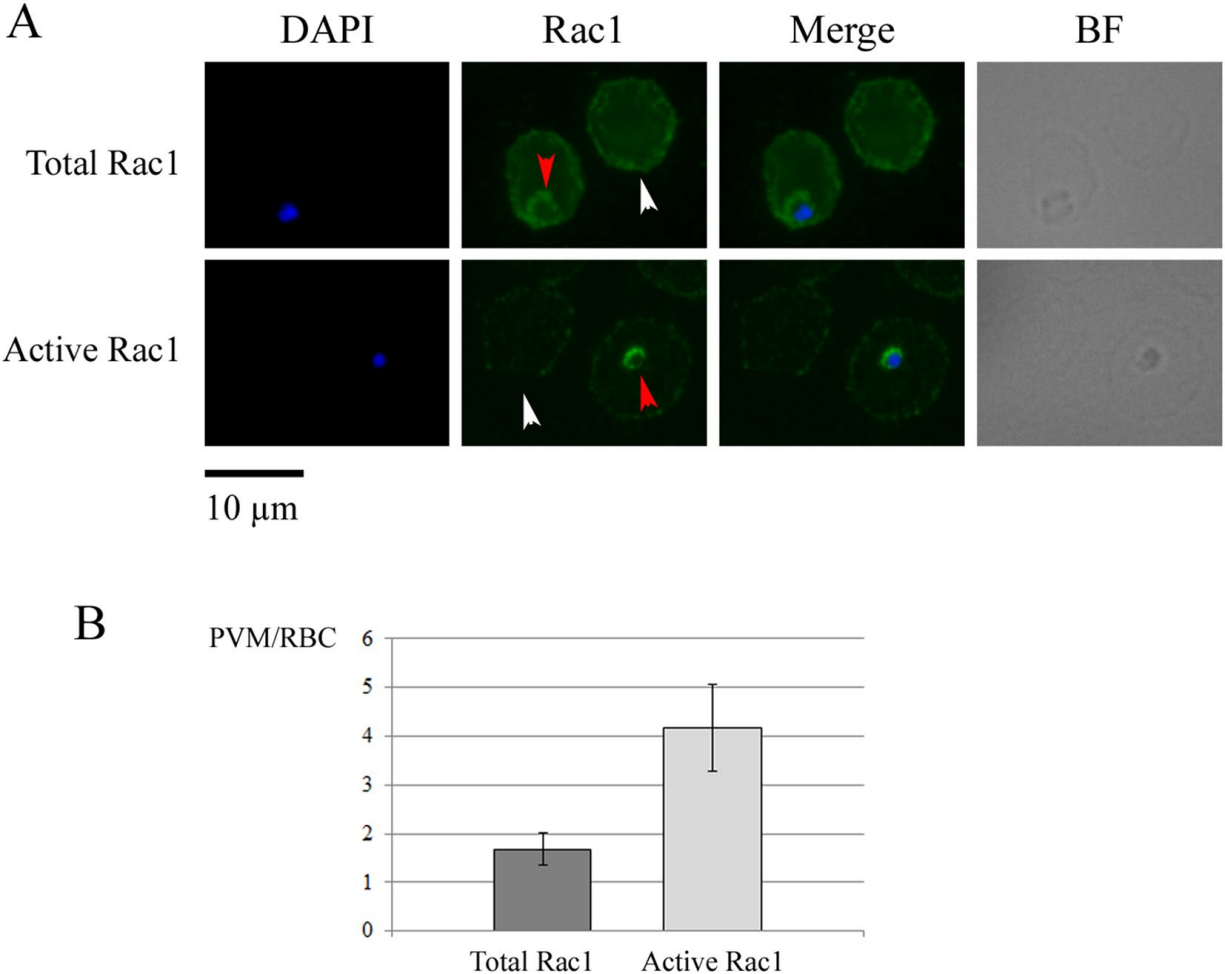
Rac1 activation state in infected erythrocytes. (**A**) IFA of an asynchronous parasite culture hybridized with anti-Rac1 (R1-ab#3) and anti-Rac1/GTP (R1-ab#6). BF: Bright field. Nuclei are stained with DAPI. Red arrows point at PVMs and white arrows point at non-parasitized erythrocytes. Scale bar: 10 µm. (**B**) Histogram showing the ratio between PVM and non-infected erythrocyte fluorescence, respectively with the anti-Rac1 (R1-ab#3) and the anti-Rac1/GTP (R1-ab#6). Student’s T-test: *P* < 10^−13^.

The activation state of the GTPase was also investigated in the intraerythrocytic stages. To do this, we performed IFAs of cultures containing mixed parasite stages with both the antibody anti-Rac1/GTP and the anti-Rac1 (Fig. 3A). The anti-Rac1/GTP fluorescence signal on uninfected erythrocytes was barely visible, while it was quite strong on the PVM. In contrast, with antibodies against total Rac1, the PVM fluorescence signal was comparable to that of uninfected RBC plasma membrane (Fig. 3A). To confirm this, we measured fluorescence intensities with the ImageJ software and calculated the PVM/non-infected RBC signal ratio with both antibodies (Fig. 3B). The ratio PVM/non-infected RBC with anti-Rac1/GTP was significantly higher compared to the same ratio with anti-Rac1 antibody. This result shows that either parasites selectively recruit active Rac1 molecules to the PVM, or promote its activation in this location.

### Rac1 role in *P. falciparum* infection

To investigate the role of Rac1 in *P. falciparum* infection, we selected two Rac1-specific inhibitors, EHT1864^34^ and 1A116^36^, based on their ability to inhibit Rac1 activity by different molecular mechanisms. EHT1864 inhibits Rac1 by acting on the catalytic site of the protein. It induces nucleotide release and prevents the binding of new nucleotides, thus keeping Rac1 in an inert state. EHT1864 does not affect Rac-GEF interaction^34^. 1A116, instead, inhibits Rac1 by binding to the protein surface containing Trp65, a residue critical for the interaction with GEFs^36^. Both inhibitors are highly selective, as they do not affect the activation state of the closely related GTPase CDC42^34,36^.

**Figure 4:**
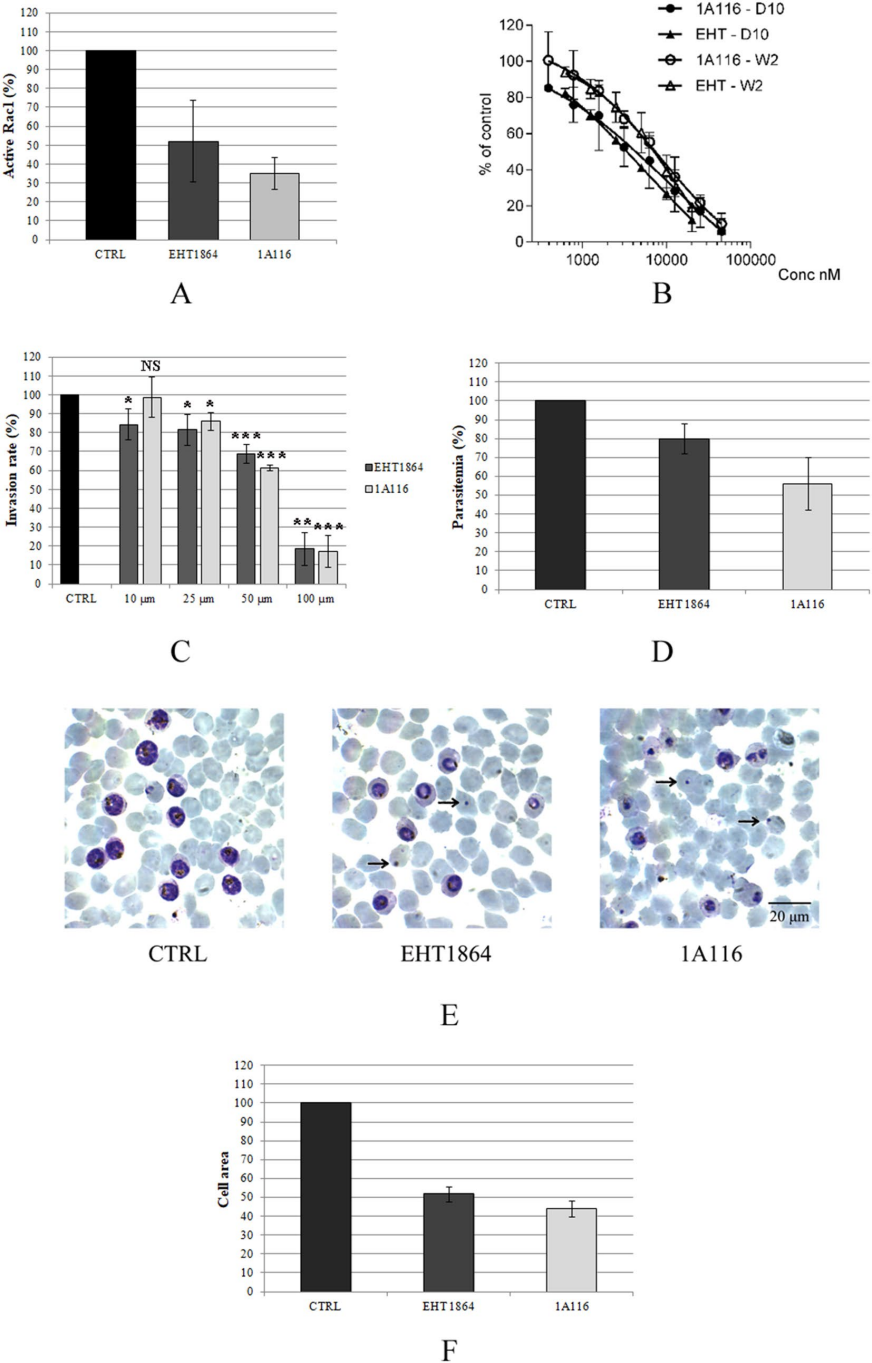
Rac1 functional analysis. (**A**) Histogram showing the G-LISA test results of erythrocytes treated with the Rac1 inhibitors EHT1864 and 1A116, as a percentage of the untreated control. Student’s T-test with both inhibitors: *P* < 0.05. (**B**) Growth curves for IC_50_ determinations. (**C**) *P. falciparum* invasion rates in the presence of the Rac1 inhibitors EHT1864 and 1A116. The number of newly infected erythrocytes at the end of the assay was normalized on the control. Data shown here are the result of three independent experiments, each performed in three technical replicates. Student’s T-test: NS: non-significant; * = * P* < 0.05; **  < 0.01; *** ≤ 0.005. (**D**) Effect of Rac1 chemical inhibitors on *P. falciparum* intraerythrocytic growth. Blood smears of a synchronous parasite culture were taken at 39 hpi, corresponding to almost mature schizont stages. Parasitemia was normalized on the control. Data shown for EHT1864 are the result of three independent biological replicates, while for 1A116 the data are the result of two biological replicates. Student’s T-test: *P* < 0.05 with both inhibitors. (**E**) Giemsa-stained smears of synchronous *P. falciparum* parasites at 39 hpi, treated with the Rac1 chemical inhibitors EHT1864 and 1A116. Arrows point at possible residues after parasite death. Scale bar: 20 µm. F. Histogram showing parasite cell areas in the inhibitor-treated samples, normalized on the untreated control. These data are the result of two biological replicates. Student’s T-test: *P* < 0.05 with both inhibitors.

We first investigated the effect of the two inhibitory compounds on Rac1 activation state in erythrocytes, since they had not previously been tested on this tissue. Rac1 activation levels were analysed in erythrocytes treated for two hours with the two inhibitors at 50 µM concentration and in the untreated control, by using the G-Lisa kit, a commercial kit specifically designed to measure the amount of active Rac1. These experiments, performed in three biological replicates, showed that the amount of active Rac1 in the treated samples was significantly lower compared to the untreated control (Fig. 4A), demonstrating that the two compounds can inhibit Rac1 activity in RBCs.

We then tested the effect of EHT1864 and 1A116 on asynchronous *P. falciparum* parasite cultures of both a strain sensitive to the common antimalarial drug chloroquine (CQ) and a resistant strain for 72 h at different concentrations. Parasite viability was assessed by measuring the parasite lactate dehydrogenase (pLDH) activity^51^, to calculate the half inhibitory concentrations (IC_50_). Both compounds showed antimalarial activity, with IC_50_ comprised between 3 and 4 µM in the CQ-sensitive strain and between 6 and 7 µM in the CQ-resistant one (Fig. 4B and Table 1).

**Table 1:** In vitro antiplasmodial activity of EHT1864 and 1A116.

	D10 IC_50_^a^ (nM)	W2 IC_50_^a^ (nM)
EHT1864	3877 ± 738	7748 ± 1821
1A116	3209 ± 1173	6888 ± 1407
Chloroquine	16 ± 3	418 ± 75

^a^The results are expressed as IC_50_ ± SD of at least three independent experiments each performed in duplicate.

To investigate more specifically the role of Rac1 in the invasion process, we tested the two compounds in invasion assays, set up as follows. Synchronous schizonts were double purified to remove any uninfected erythrocyte and allowed to reinvade new RBCs in the presence of EHT1864 and 1A116, at concentrations ranging from 10 to 100 µM. The number of new infections was estimated by FACS analysis and expressed as a percentage of the invasion rate in the untreated control. Both compounds significantly inhibited parasite invasion of the host cell in a dose-dependent manner (Fig. 4C). These results suggest a role of Rac1 in *P. falciparum* invasion and indicate that parasites may exploit this erythrocyte GTPase to enter the host cell.

Once investigated Rac1 role in *P. falciparum* invasion of human erythrocytes, we sought to assess whether the GTPase also plays a role in the subsequent intraerythrocytic parasite growth. To do this, synchronous schizonts were purified by 60% Percoll gradient, then seeded on fresh human RBCs and allowed to invade the host cells for two hours. Schizonts were then killed by 5% sorbitol treatment, leaving in culture only young stage parasites, born within the last two hours^52^. Synchronous parasites were then cultured in the presence of the two Rac1 chemical inhibitors EHT1864 and 1A116 at concentrations corresponding to ten-fold the IC_50_ concentrations previously assessed. Blood smears were taken at 39 hpi, when parasites were almost mature, but none of them had yet released daughter merozoites. Parasitemia was assessed by counting parasites on Giemsa stained smears. These experiments showed a trend of parasitemia decrease in the inhibitor-treated samples compared to the control, more evident for 1A116 (Fig. 4D). In the inhibitor-treated samples, the surviving parasites were likewise smaller, compared to the control, and showed an aberrant morphology (Fig. 4E,F). Consistent with this, we also observed the appearance of dots inside some erythrocytes, possibly representing parasite residues after death by shrinkage. These experiments showed that the two Rac1 chemical inhibitors affect *P. falciparum* intraerythrocytic growth, revealing a possible role for the GTPase also in this phase of the asexual cycle.

## Discussion

We here showed that erythrocyte treatment with two Rac1 inhibitors significantly reduced parasite invasion rates. Being Rac1 the only Rac protein identified by western blot in mature erythrocytes and being Rac1 homologues absent in the parasite genome, the inhibitors were probably acting specifically on this GTPase, even though we cannot completely rule out possible non-specific toxic effects.

Nevertheless, the fact that the two inhibitory Rac1 compounds have completely different chemical structures and different inhibition mechanisms, and that invasion rate reductions had a dose-dependent profile in both cases, is suggestive of a specific effect on the GTPase.

Since treatment with Rac1 inhibitors did not induce a complete inhibition of parasite invasion, we cannot rule out that parasites may use alternative invasion pathways or that the GTPase might just have a secondary role in the invasion process. However, our data claim for the first time a role of Rac1 in *P. falciparum* invasion of host erythrocytes. This result enforces the concept firstly suggested by Harrison et al*.* that parasites exploit the host signal transduction machinery to invade erythrocytes^8^.

Intracellular pathogen entry into the host cell is strictly associated with their ability to manipulate the host actin cytoskeleton^18,20,21^. Also intracellular apicomplexan parasites, such as *Cryptosporidium parvum*, *T. gondii* and *P. berghei* require host actin polymerization in order to enter inside the host cell^53,54^. On the other hand, a recent work from Zuccala et al. showed that treatment with reversible actin polymerization inhibitors does not prevent *P. falciparum* entry into the host cell. The authors state that the participation of host cell actin to *P. falciparum* invasion appears unlikely, even though they cannot definitely rule it out^55^. Since Rac1 main biological role is actin cytoskeleton modulation, our results tend to contradict these conclusions and on the contrary support a role of the host actin in *P. falciparum* invasion, as previously reported for other apicomplexan parasites^53,54^.

After invasion, Rac1 is recruited to the PVM and gradually depleted from the erythrocyte membrane. This result is consistent with previous findings showing by comparative proteomics that Rac1 is between the proteins depleted from the infected erythrocyte membrane^56^. It was suggested that Rac1 removal from erythrocyte membrane may contribute to the host cytoskeleton dismantling to allow daughter cells release^56^. We also demonstrated that Rac1 on the PVM is mostly in the active form. Based on available information, we cannot definitely assess why parasites induce Rac1 activation in this phase of the life cycle, but we hypothesize it may be related to actin cytoskeleton modulation to facilitate parasite volume increase. It has previously been reported that host actin plays a role in *P. berghei* intracellular growth inside hepatocytes, possibly involving PVM remodelling. It was reported that hepatocyte actin accumulates around the PVM and important actin reorganization events take place during parasite growth^57^. A recent work demonstrated that this is the case also in erythrocytes infected by *P. falciparum*^58^. Our results support these reports. The volume reduction of parasites treated with Rac1 inhibitors could be related to Rac1 role in PVM remodelling, whose inhibition may cause parasite shrinkage and eventually death.

Finally, Rac1 could also play a role in intracellular vesicle trafficking. In fact, actin is known to be involved in intracellular trafficking of *Maurer’s clefts*, parasite-induced membranous compartments that *P. falciparum* forms inside the host erythrocyte to export factors involved in cytoadherence on the surface of the host cell^59^. Rac1 chemical inhibitors could be more effective in vivo, by acting on *Maurer’s clefts* trafficking and impairing RBCs cytoadherence, which is the main cause of severe clinical symptoms in *P. falciparum* infections.

In conclusion, we here propose the human GTPase Rac1 as a promising target for the development of novel antimalarial drugs addressed against the host, even though other studies will be needed to definitely demonstrate that Rac1 plays a role in *P. falciparum invasion*. Since reverse genetics cannot be achieved in mature erythrocytes, a possible strategy could be to load erythrocytes with a dominant-negative mutant Rac1^60^.

Targeting host molecules that pathogens exploit to enter or develop inside the host cell is a novel strategy to limit insurgence of drug-resistance. This approach is less likely to generate drug resistance than conventional antimicrobial therapies, since the pathogen needs to redirect its entire infection strategy to compensate for a missing interaction with a host molecule, and could possibly be effective also against different *P. falciparum* strains^61–64^. Host-targeted molecules have already been proven effective against different infectious diseases^65–67^. Some host-targeted compounds are already available for clinical use^68,69^. In malaria, conoidin A, an irreversible inhibitor of the host peroxidase Prx2, prevents growth of *P. falciparum* and makes it more sensitive to chloroquine^70^.

Rac1 has several advantages as a pharmacological target, compared to other molecules. Due to its role in several pathologies and cellular mechanisms, it is a widely studied subject: more than 7.500 articles have been published about this GTPase. Rac1 crystal structure, as well as of several of its interacting proteins are known and numerous Rac1 inhibitors are already available. Among these inhibitors, some have already been tested in vivo^71^ and others are already in clinical use^72^. The growing collection of Rac1 inhibitory compounds could be repurposed for malaria.

Due to the presence of Rac1 in several human tissues and its involvement in many molecular pathways, therapies targeting this protein might have toxic effects. Strategies to overcome this issue should be explored, such as specifically addressing inhibitory compounds to human erythrocytes by tissue-specific drug delivery strategies. None of Rac1 interactors was identified in mature erythrocytes by proteomic studies. Other studies will be necessary to better understand the mechanisms by which parasites exploit Rac1 signalling, to possibly identify specific Rac1 interactors in erythrocytes, as potential additional antimalarial drug targets.

Nevertheless, this study contributed to the identification of a novel erythrocyte protein possibly exploited by malaria parasites during invasion of the host cell.

## Materials and methods

### Sample preparation and Western blot

Whole blood collected from 7 donors was washed in RPMI 4 times in order to remove plasma, platelets and leucocytes. Any residual leucocyte was removed by filtration with Plasmodipur filters (Europroxima) and erythrocyte purity was confirmed by flow cytometry (Gallios Flow cytometer equipped with 3 lasers: 405 nm, 488 nm, 633 nm; Beckman Coulter). In detail, blood samples were stained with anti-CD45-PB (BD bioscience), anti-CD16-FITC (BD bioscience) to identify granulocytes and anti-CD61-PE (eBioscence), anti-CD41-FITC (eBioscience) to identify platelets. The number of erythrocytes was determined by adding cell counting beads to flow cytometric sample (Thermo Fisher). Data were analysed by using Kaluza Analysis Software (Beckman Coulter). Purified erythrocyte membranes were obtained by RBC lysis in hypotonic lysis buffer (5 mM Na-phosphate, 0.5 mM EDTA, pH 8), followed by 4 washings to eliminate the soluble fraction.

For western blot analysis, erythrocyte membranes were lysed in Pierce IP Lysis Buffer (Thermo Fisher) for 20 min at 4 °C. The sample was then centrifuged at 16.000 g for 10 min and supernatant was separated from pellet. Purified GST-tagged Rac1 protein (Cytoskeleton) was used to show the specificity of the antibody against Rac2. Purified His-tagged Rac2 protein (Sino Biological) was used as a positive control. All samples were then subjected to SDS-PAGE under reducing conditions, followed by transfer to nitrocellulose membrane (Sartorius). Anti-Rac1 antibodies used are shown in Table S1. Dilutions: anti-Rac mouse monoclonal (R1-ab#1) 1:1000, anti-Rac1 mouse monoclonal (R1- ab#2) and anti Rac-2 rabbit polyclonal (ThermoFisher) 1:1000. The filters were then incubated with anti-mouse or anti-rabbit HRP-conjugated antibodies (Pierce) and the immunocomplexes were visualized using chemioluminescence ECL detection system (Millipore).

### Immuno-fluorescence assays

Blood smears from parasite cultures (3D7 strain) were fixed in 4% paraformaldehyde/0.015% glutaraldehyde for 30 min at room temperature. Cells were then permeabilized with 0,1% Triton-X100 in PBS for 10 min and incubated for 1 h with the following primary antibodies (see Table S1): Rac1 mouse monoclonal antibody (R1-ab#3) at 1:200 dilution, anti-Rac1/GTP mouse monoclonal antibody (R1-ab#6) 1:200 dilution, anti-Rac1 rabbit polyclonal antibody (R1-ab#4) 1:20 dilution, N201 mouse polyclonal antibody^50^ 1:100 dilution, RON4 mouse polyclonal antibody^48^ 1:100 dilution, anti-Band3 mouse monoclonal antibody (Sigma) 1:200 dilution, anti-AMA1 rabbit polyclonal antibody^73^ 1:100 dilution, anti-MSP1 rabbit polyclonal antibody^74^ 1:100 dilution. Samples were then washed in PBS and incubated with the secondary antibodies: anti-mouse and anti-rabbit fluorescein (Invitrogen and ThermoFisher respectively) 1:200 dilution, anti-mouse and anti-rabbit rhodamine (ThermoFisher) 1:200 dilution and with the nuclear marker DAPI (Life Technologies) 500 nG/ml. Samples were washed again in PBS and smears were mounted in Vectashield (Vector Laboratories). Negative controls without primary antibodies have been performed, resulting in complete absence of fluorescence signals.

Smears were then analysed with a Leitz DMRB microscope, equipped with BP 340–380, BP 470–490 and BP 515–560 filters to visualize respectively DAPI, fluorescein and rhodamine fluorescence. The same fields were then observed in bright light and acquired with a Leica DFC340-FX camera. Images were analyzed with LAS (v 3.8, Leica Microsystems) software. At least 200 cells were observed in each immuno-fluorescence assay. Images were analysed by ImageJ, using the plug-in Coloc2 for co-localization experiments and the “grey scale media” to measure fluorescence. For co-localization analysis, three biological replicates were taken into account and at least 30 cells were analyzed in each replicate, in order to calculate Pearson’s correlation coefficient.

### Parasite cultures

Cultures of CQ-sensitive D10, CQ-resistant W2 and 3D7 strains were maintained according to Trager and Jensen with slight modifications^75^. Briefly, all the strains were cultured at 5% haematocrit (human type A-positive RBCs for D10 and W2; 0-positive RBCs for 3D7) in complete medium at 37 °C in a standard gas mixture consisting of 1% O_2_, 5% CO_2,_ 94% N_2_. Complete medium is made up of RPMI 1640 (EuroClone, Celbio, Gibco) with the addition of 0.01% hypoxanthine (Sigma-Aldrich), 20 mM Hepes (Euroclone), 2 mM glutamine (Euroclone). All the parasites were cultured in the presence of 1% AlbuMaxII (lipid-rich bovine serum albumin, Life Technologies) (D10 and W2), or cultured in the presence of 10% naturally-clotted heat-inactivated 0 + human serum (Interstate Blood Bank, Inc.) (3D7).

### Rac1 activation assay

Whole blood collected from 7 donors was washed in RPMI 4 times in order to remove plasma, platelets and leucocytes. The number of erythrocytes was determined by using a cell counting chamber. Erythrocytes were treated for two hours at 37° C with or without Rac1 chemical inhibitors and then washed. In each sample, Rac1 activation state was measured with the G-LISA Rac1 Activation Assay Biochem Kit—Luminescence Based (Cytoskeleton), an ELISA based assays that measures the amount of Rac1 active form in cell lysates. Luminescence intensity was assessed with a Biotek Sinergy HT plate reader (Biotek). EHT1863 was purchased by Santa Cruz Biotechnologes, 1A116 was kindly provided by Prof. Daniel Gomez, Universidad Nacional de Quilmes, Buenos Aires, Argentina.

### Antiplasmodial activity assays

Compounds were dissolved in DMSO and then diluted with culture medium to achieve the required concentrations (final DMSO concentration < 1%, which is nontoxic to the parasite). Drugs were placed in 96 well flat-bottom microplates and serial dilutions made. Asynchronous cultures (D10 and W2 strains) with parasitemia of 1–1.5% and 1% final haematocrit were aliquoted into the plates and incubated for 72 h at 37 °C. Parasite growth was determined spectrophotometrically (OD_650_) by measuring the activity of the parasite lactate dehydrogenase (pLDH), according to a modified version of Makler's method in control and drug-treated cultures^51^. Antiplasmodial activity is expressed as the 50% inhibitory concentrations (IC_50_). The IC_50_ was extrapolated from the non-linear regression analysis of the concentration–response curve using the Gene5 software. Each IC_50_ value is the mean ± standard deviation of at least three separate experiments performed in duplicate.

### Invasion assays

Parasites (3D7 strain) were pre-synchronized 44 h before the assay by 60% Percoll gradient^76^. Just before the invasion assay, cultures were double purified by 60% Percoll gradient and MACS column magnetic separation system (Miltenyi Biotec), in order to remove any uninfected erythrocytes^77^. Purified schizonts were allowed to reinvade human RBCs for 6 h in a 96-well plate (1% hematocrit, at a parasitemia ranging from 2 to 8%). Erythrocytes were pretreated for two hours with the inhibitors, before adding the schizonts. A T0 sample was fixed just after schizont seeding. All samples were fixed in 4% paraformaldehyde/0.075% glutaraldehyde for 30 min at room temperature, then washed in PBS. After fixation, parasite nuclei were stained with 2 µM Hoechst 33,342 (Thermo Fisher) for 30 min at 37 °C in the dark. Cells were then washed in PBS and counted by using a GALLIOS cytometer (Beckman Coulter), acquiring 200,000 events per sample. Initial gating was carried out with uninfected erythrocytes to exclude possible erythrocyte autofluorescence. Invasion rates were calculated by subtracting the number of schizonts at the end of the assay (6 hpi) from the number of schizonts in the T0 sample, thus obtaining the number of merozoite-releasing schizonts. Then, the number of new infections was normalized on the number of merozoite-releasing schizonts, to obtain the parasitized erythrocyte multiplication rate (PEMR). This value was finally normalized on the control sample, thus obtaining the invasion rate value.

### Growth assays

Parasites (3D7 strain) were pre-synchronized 44 h before the assay by 60% Percoll gradient^76^. Just before the beginning of the assay, schizonts were purified by 60% Percoll gradient and allowed to invade fresh human RBCs for two hours in a 24-well plate in complete medium. The culture was then treated with 5% sorbitol to kill all parasites except young stages resulting from the recent invasion^52^. Cells were then put back in culture in the presence of Rac1 chemical inhibitors. Blood smears were taken at 39 hpi, corresponding to schizont stage, and then Giemsa-stained and counted by optical microscopy in order to assess parasitemia. Parasite cell areas of 30 cells for each biological replicate were measured by using the software ImageJ.

### Ethics statement

Blood for analyzing erythrocyte proteins and for propagating *P. falciparum* cultures was obtained from the Transfusion Center of Policlinico Umberto I, directed by Prof. Gabriella Girelli. The experimental protocol was approved by the “Policlinico Umberto I Ethics Committee”. Blood samples were screened for known pathogens in accordance with the Italian National Regulations. No information about the donor, other than the blood group was obtained or recorded by the user. A written informed consent was asked to blood donors, including a statement that participation was voluntary. No minors were included in this study.

All handling of biological material and the whole experimental procedure was performed under strict safety procedures, in compliance with national (Ministry of Health-Decree of 3rd of March 2005, Official Gazette n. 85, 13-4-2005) and EU regulations on suitability assessment of blood donors and blood components.

## Supplementary Information

Supplementary Information.
