# Supplementary material for: Characterization of the erythrocyte GTPase Rac1 in relation to Plasmodium falciparum invasion
Source: Sci Rep. 2020 Dec 16;10:22054. doi: 10.1038/s41598-020-79052-0 (PMC7744522; doi:10.1038/s41598-020-79052-0)
Supplement: Supplementary file 1 — Supplementary Information. [file 41598_2020_79052_MOESM1_ESM.docx]

**Characterization of the erythrocyte GTPase Rac1 in relation to *Plasmodium falciparum* invasion**

Silvio Paone^1,2^, Sarah D’Alessandro^3#^, Silvia Parapini^4#^, Francesco Celani^1^, Valentina Tirelli^1^, Manoochehr Pourshaban^1^ and Anna Olivieri^1^*.

1: Istituto Superiore di Sanità, Dipartimento di Malattie Infettive, Rome, Italy.

2: Sapienza University of Rome, Dipartimento di Sanità Pubblica e Malattie Infettive, Rome, Italy.

3: University of Milan, Dipartimento di Scienze Biomediche, Chirurgiche e Odontoiatriche, Milan, Italy

4: University of Milan, Dipartimento di Scienze Biomediche per la Salute, Milan, Italy

^#^ These authors contributed equally to the work.

* corresponding author. Email: anna.olivieri@iss.it


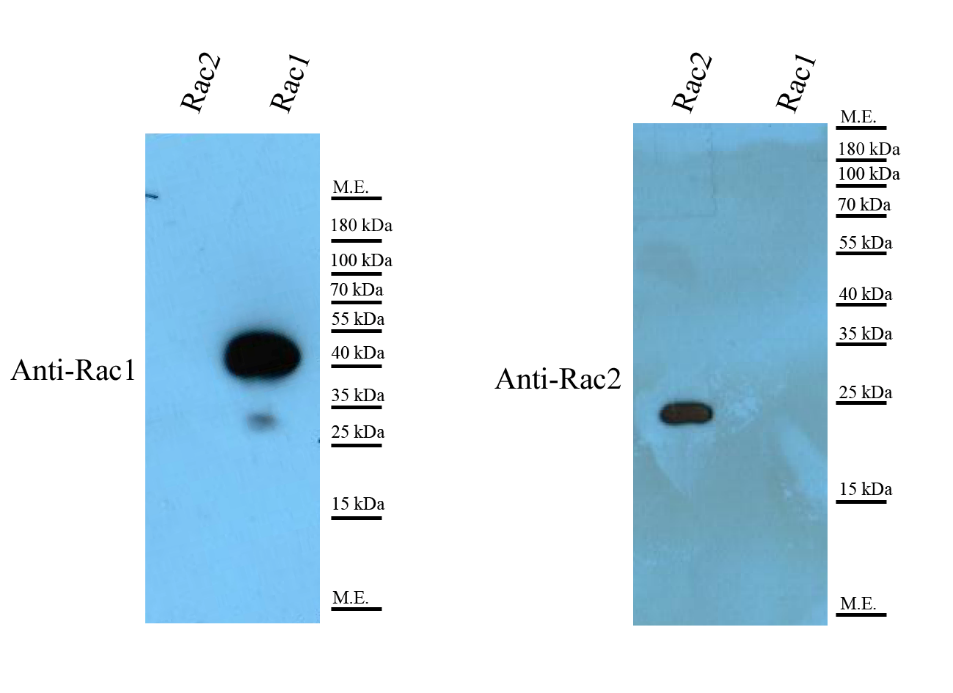


**Figure S1.** **Antibody validation.** Left panel: 233 ng of purified Rac1-GST protein, corresponding to 100 nG of Rac1, and 100 ng of purified Rac2-His protein were probed with the anti-Rac1 specific antibody (R1-ab#2). The signal of Rac1-GST protein was detected at about 50 kDa, as expected. Right panel: in a different gel, same amounts of purified proteins were probed with the anti-Rac2 specific antibody. The signal of Rac2-His was detected at about 25 kDa, as expected. The anti-Rac1 antibody did not give any signal corresponding to the Rac2-His protein and the anti-Rac2 antibody did not recognize the anti-Rac1-GST protein. This experiment confirmed that the antibodies are selective for the Rac protein they were raised against. M.E. Membrane edges.


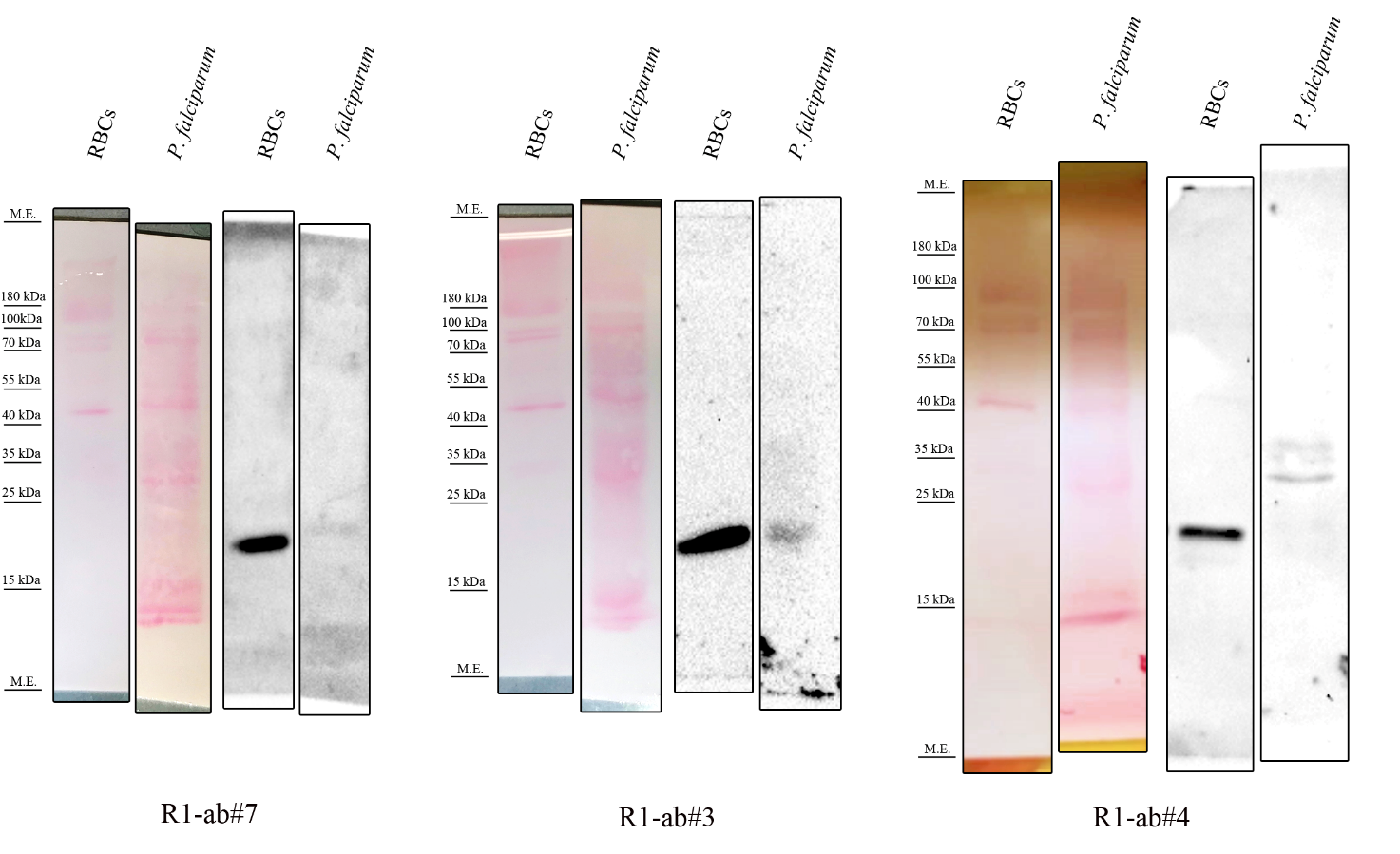


**Figure S2. Antibody validation.** Western blot of protein lysates from 5x10^7^ human erythrocyte membranes (RBCs) and a comparable amount of protein lysates from saponin-treated *P. falciparum* asexual parasites, as shown by Ponceau-S stained filters. Parasites were treated with 0.15% saponin in PBS at 4°C for 5 minutes and then centrifuged at 13.000 g at 4°C for 15 minutes. After removal of the supernatant, the sample was washed once in PBS and then lysed in protein loading buffer at 95°C for 5 minutes. Filters were hybridized with three different Rac1 antibodies: R1-ab#7 (1:5000 dilution), R1-ab#3 (1:500 dilution) and R1-ab#4 (1:500 dilution) in Table S1. In RBC lanes, a signal corresponding to Rac1 size, at about 21 kDa, is visible with all antibodies. R1-ab#7 and R1-ab#4 also show very faint signals in the lanes of *P. falciparum* purified parasites, possibly resulting from low amounts of Rac1 on residual erythrocyte membranes still present in the sample after saponin-treatment. No signal corresponding to Rac1 size was detected in the lane of *P. falciparum* purified parasites hybridized with R1-ab#3 and only a weak spurious band at around 30 kDa was visible. All images were acquired with same exposure time and gain. M.E. Membrane edges.


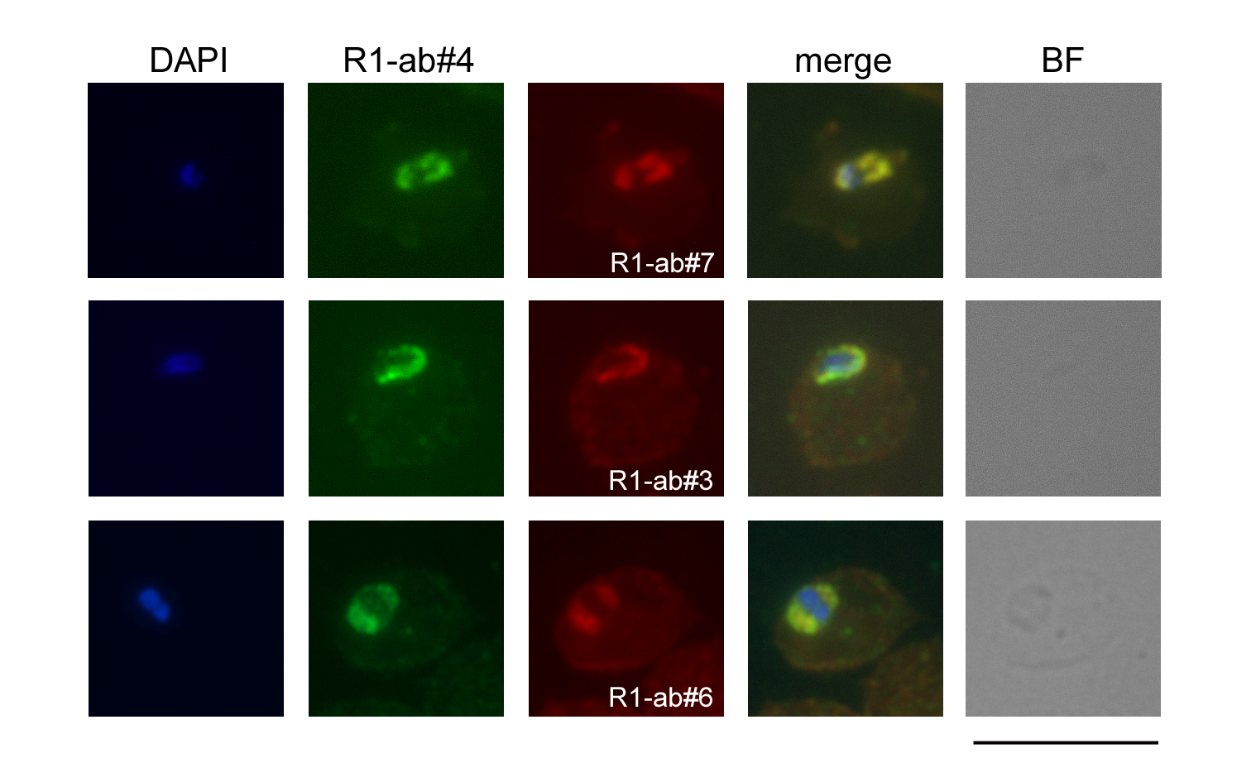


**Figure S3. Co-localization of anti-Rac1 antibodies.** IFA of asynchronous parasite stages with four anti-Rac1 commercial antibodies, were performed on mixed parasite cultures, showing that they all co-localize with each other in all the different parasite stages. The figure only shows a single parasite stage as an example. Anti-Rac1 rabbit monoclonal antibody R1-ab#7 was used at 1:20 dilution. Nuclei are stained with DAPI. BF: bright field. Scale bar: 10 µm.


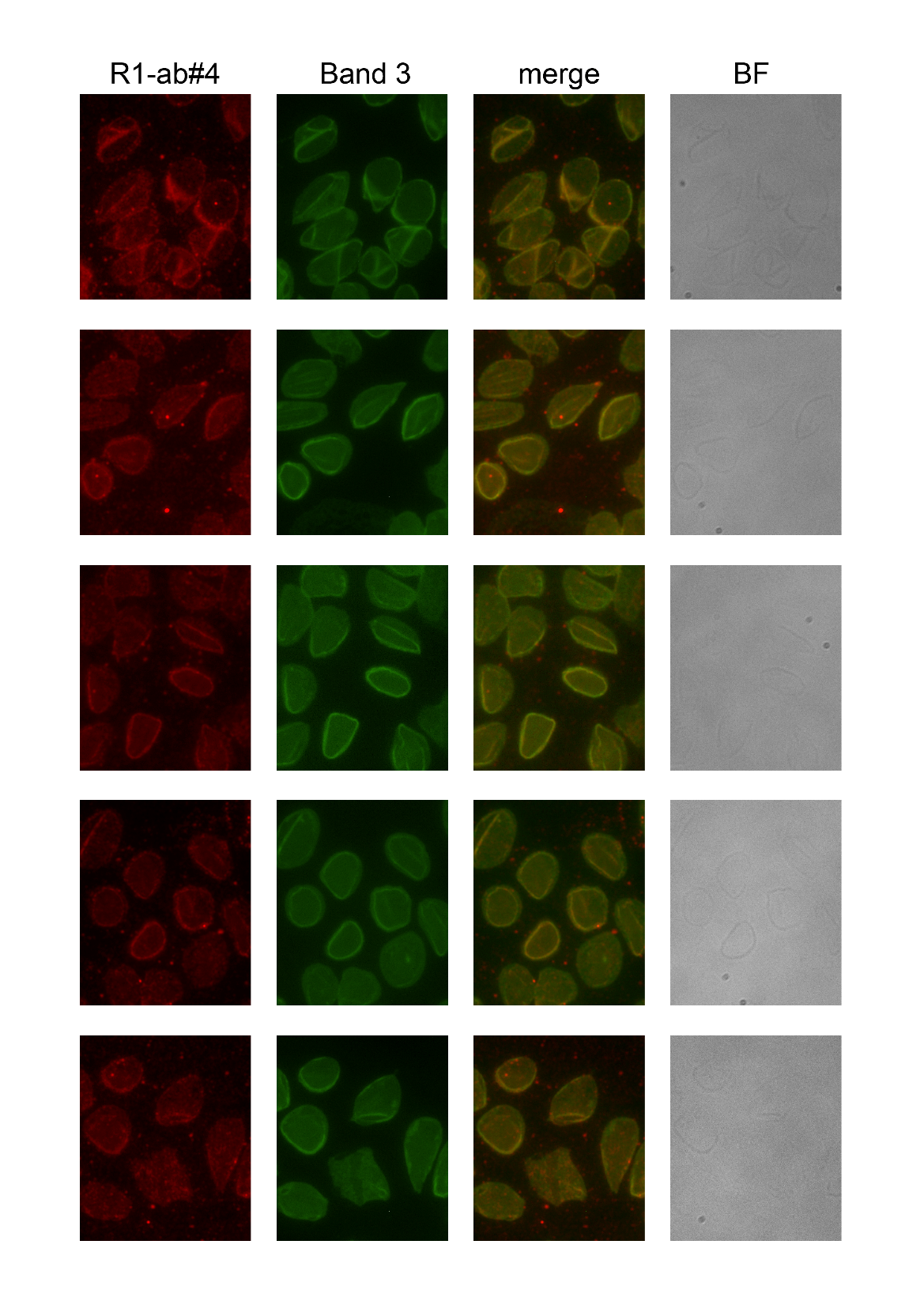


**Figure S4. Rac1 localization in erythrocytes.** IFAs of uninfected human erythrocytes with anti-Rac1(R1-ab#4) and anti- Band3, used as a marker of the erythrocyte membrane. BF: bright field. Scale bar: 10 µm.


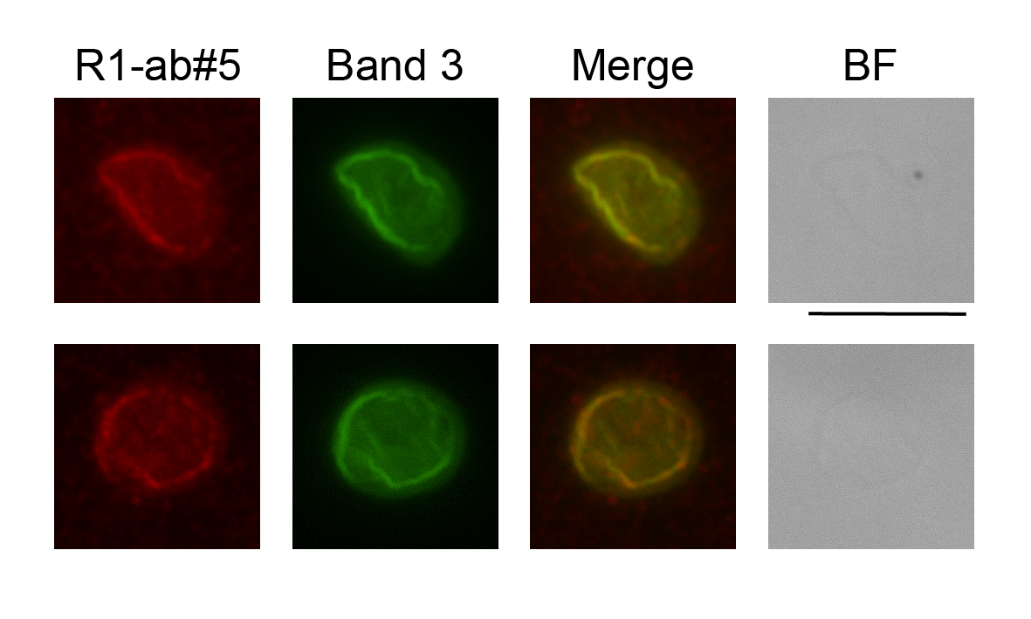


**Figure S5. Rac1 localization in erythrocytes.** IFAs of uninfected human erythrocytes with anti-Rac1(R1-ab#5), used at 1:20 dilution, and anti-Band3, used as a marker of the erythrocyte membrane. BF: bright field. Scale bar: 10 µm.


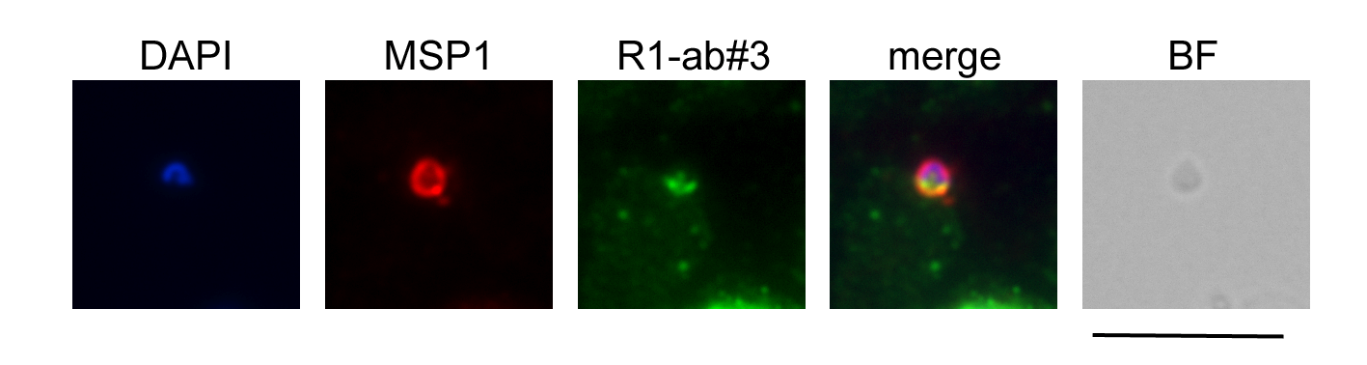


**Figure S6. Rac1 subcellular localization upon invasion.** IFA of invading parasite with anti-Rac1 (R1-ab#3). Anti-MSP1 antibody was used as a marker of merozoite surface. BF: Bright field. Nuclei are stained with DAPI. Scale bar: 10 µm.


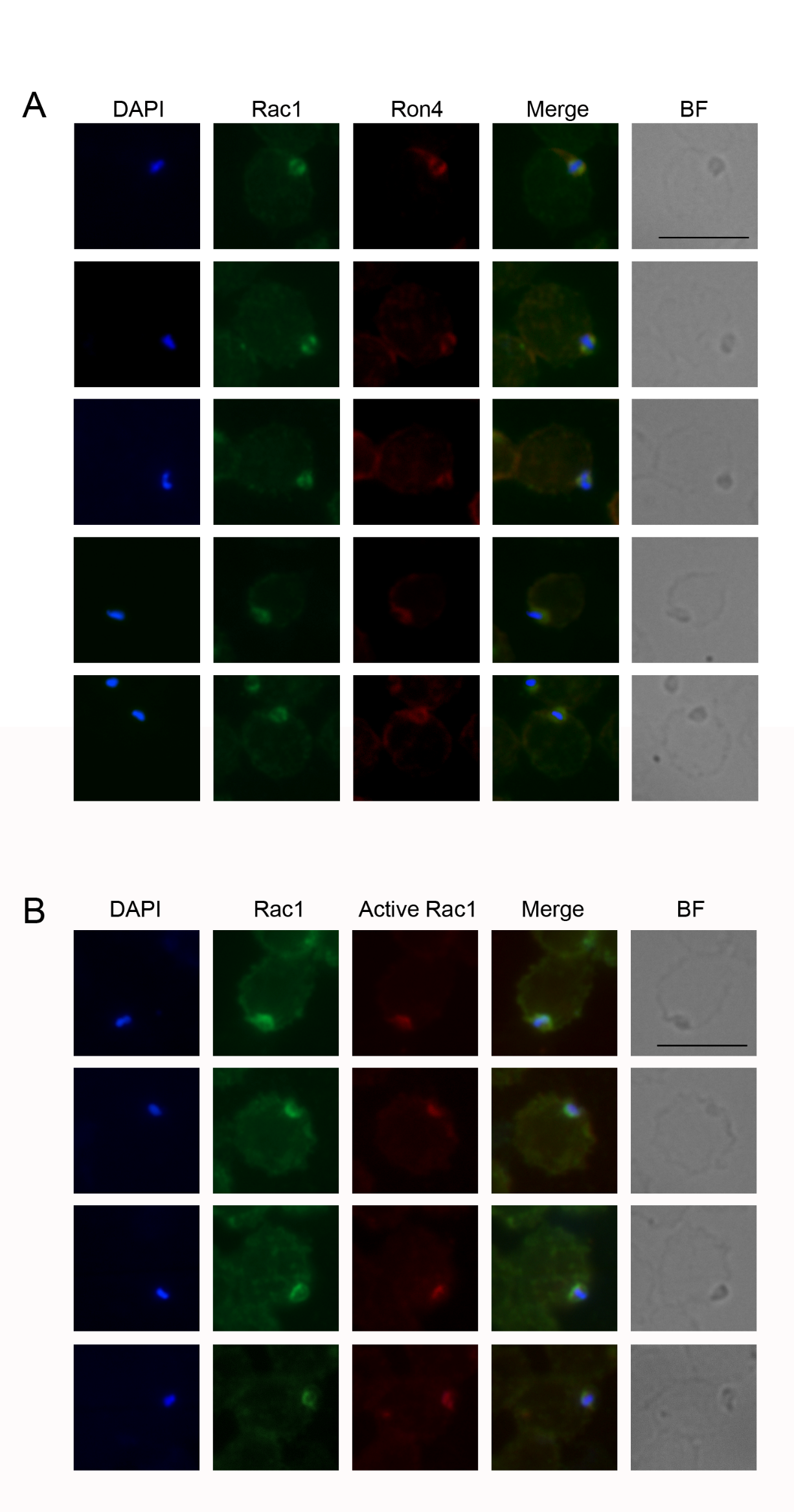


**Figure S7. Rac1 subcellular localization upon invasion.** A. IFA of invading parasites with the polyclonal anti-Rac1 R1-ab#4. Most of the nuclei show a typical bilobed shape. Anti-RON4 antibody was used as a marker of the moving junction. BF: Bright field. Nuclei are stained with DAPI. Scale bar: 10 µm. B. IFA of invading parasites with anti-Rac1 R1-ab#4 and anti-Rac1/GTP (R1-ab#6). BF: Bright field. Nuclei are stained with DAPI. Scale bar: 10 µm.


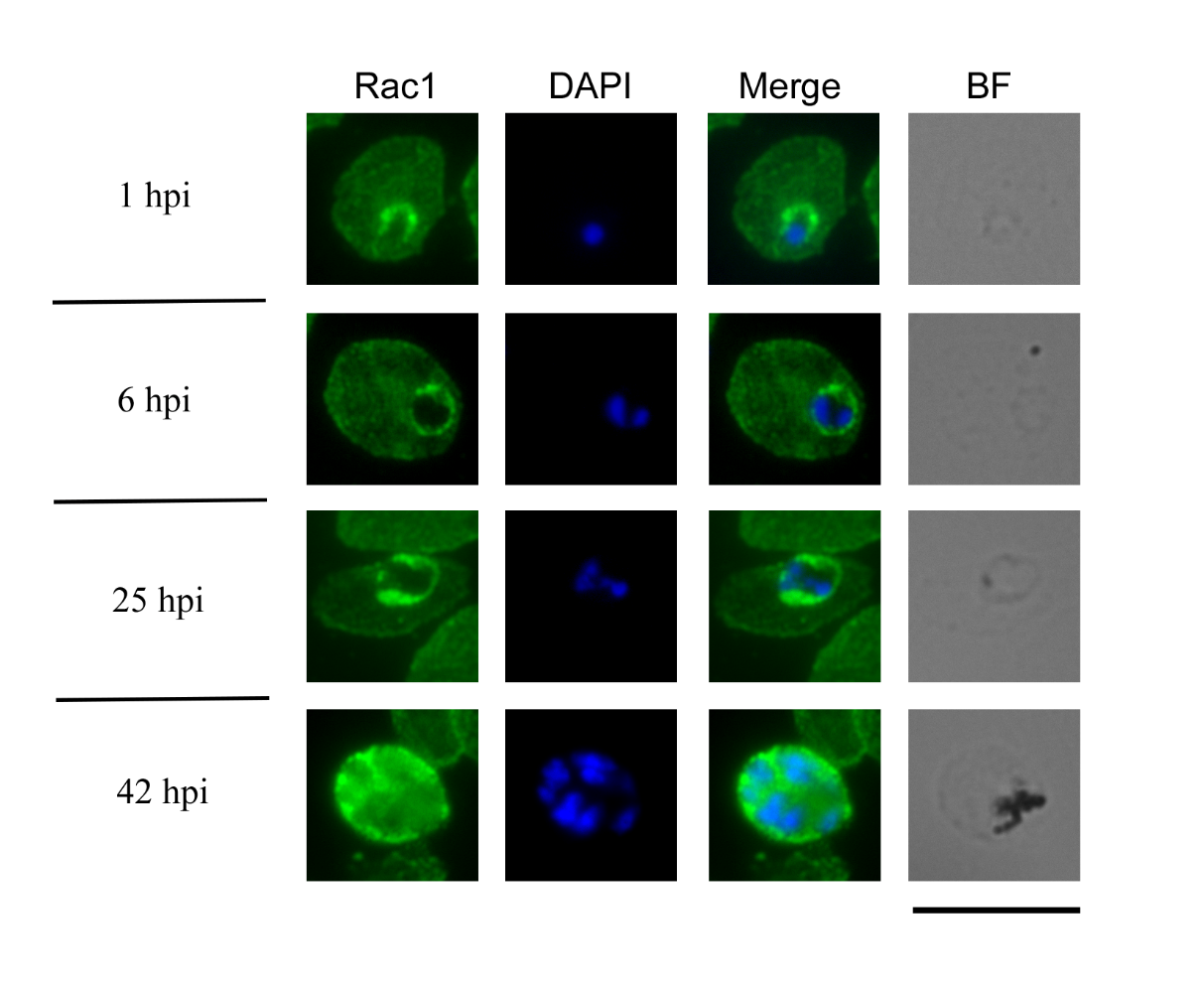


**Figure S8. Rac1 localization in infected erythrocytes.** IFAs of synchronous *P. falciparum* parasite stages at 1 hpi, 6 hpi, 25 hpi and 42 hpi with the monoclonal anti-Rac1 antibody R1-ab#3. Different exposure times were used in each image. BF: bright field. Scale bar: 10 µm.


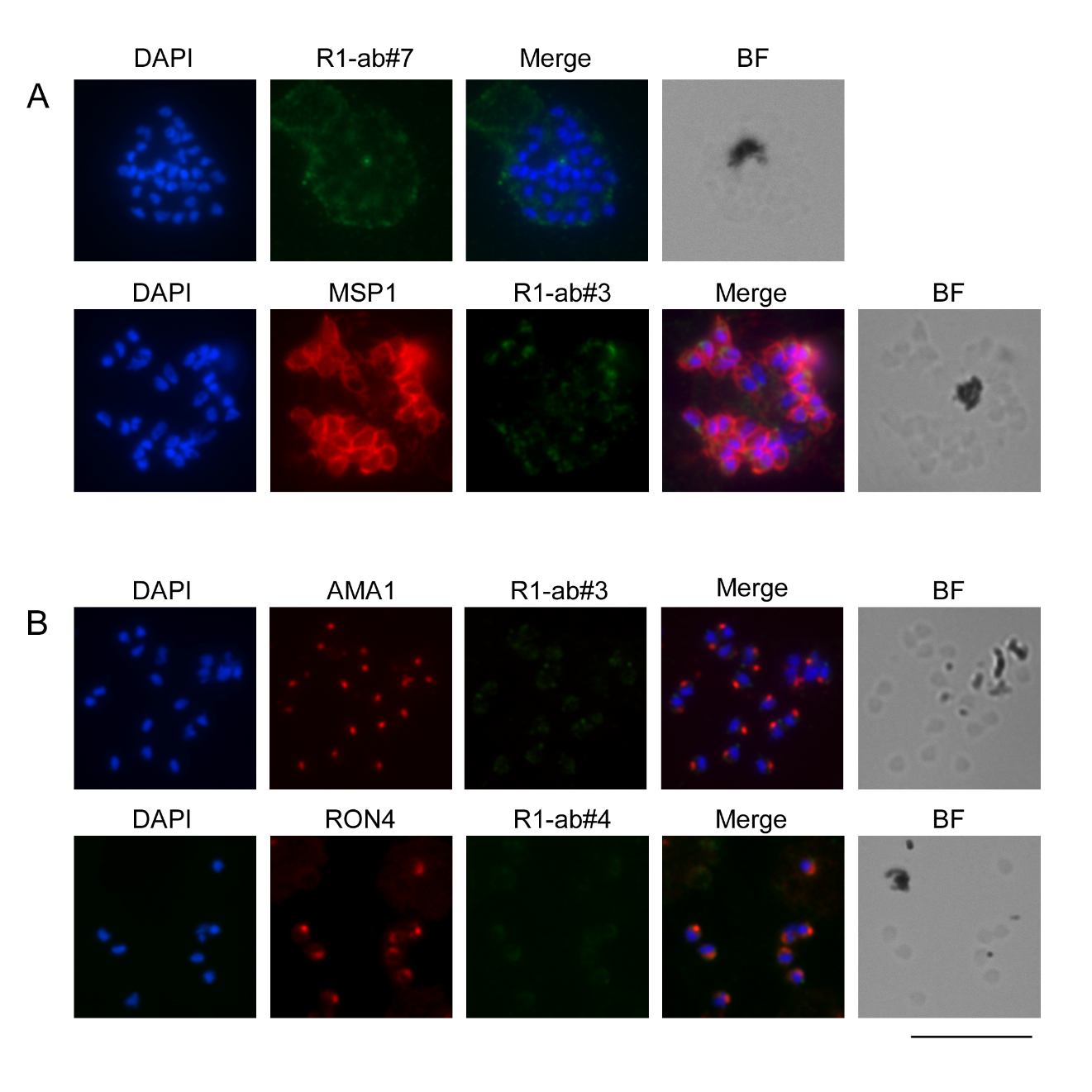


**Figure S9. Rac1 localization in mature schizonts and merozoites.** A. IFA of mature schizonts with anti-Rac1 R1-ab#7 (upper panel) and R1-ab#3 (lower panel). MSP1 was used as a marker of merozoite surface. B. IFA of free *P. falciparum* merozoites with R1-ab#3 (upper panel) and R1-ab#4 (lower panel). Apical Membrane Antigen 1 (AMA1) was used as a marker of micronemes (1) and RON4 as a rhoptry marker. Nuclei are stained with DAPI. BF: bright field. Scale bar: 10 µm.


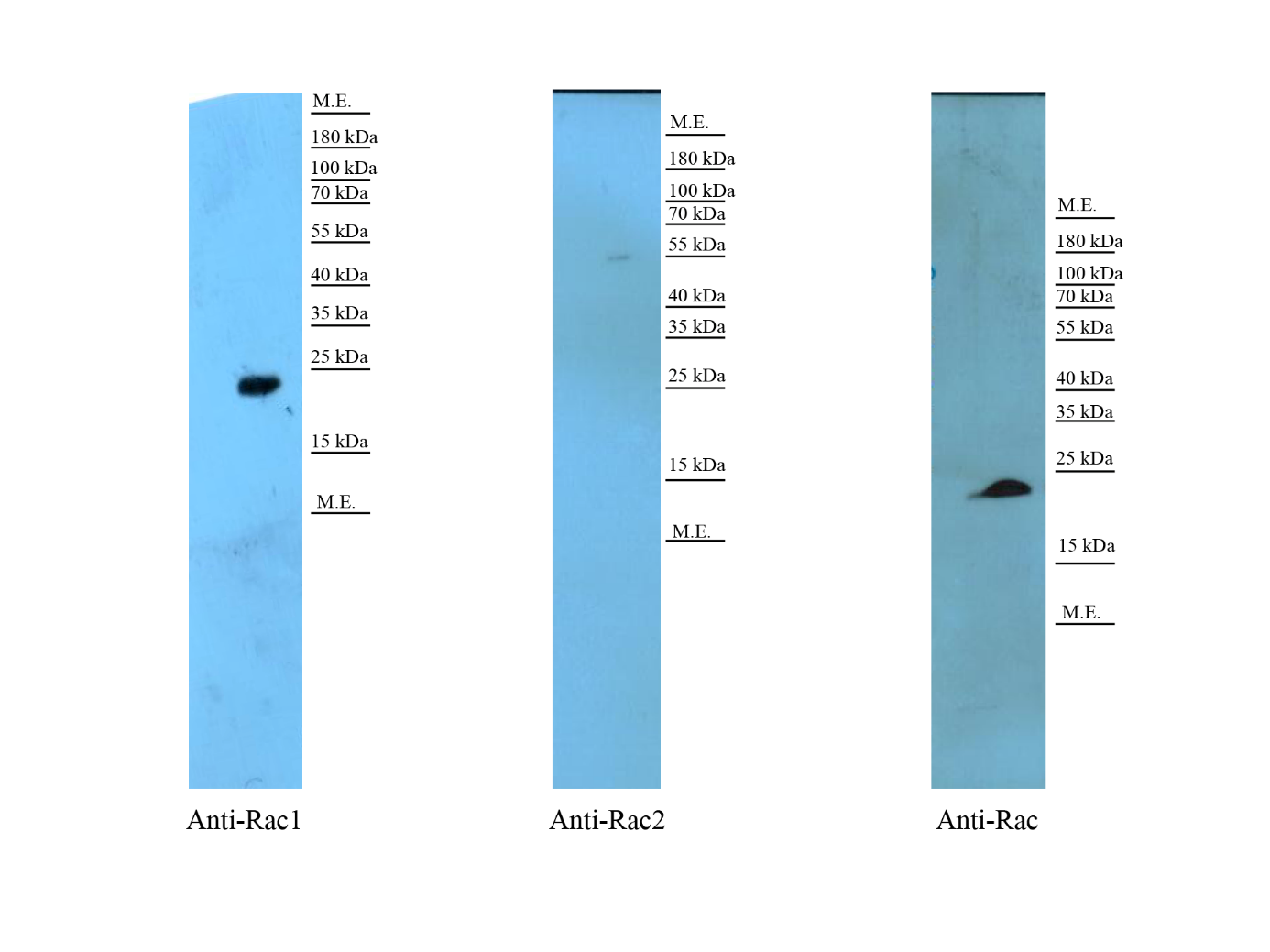


**Figure S10.** **Full lanes from Fig. 1.** Full lanes of western blot experiments shown in figure 1. M.E. Membrane edges.

| Antibody code | Target | Host | Type | Brand |
| --- | --- | --- | --- | --- |
| R1-ab#1 | Rac1/2/3 | Mouse | Monoclonal | Santa Cruz |
| R1-ab#2 | Rac1 | Mouse | Monoclonal | Cytoskeleton |
| R1-ab#3 | Rac1 | Mouse | Monoclonal | ProteinTech |
| R1-ab#4 | Rac1 | Rabbit | Polyclonal | ProteinTech |
| R1-ab#5 | Rac1 | Rabbit | Polyclonal | ThermoFisher |
| R1-ab#6 | Rac1/GTP | Mouse | Monoclonal | NewEast Bio |
| R1-ab#7 | Rac1 | Mouse | Monoclonal | Millipore |

Table S1. List of all the anti-Rac1 antibodies used.

1. C. G. Donahue, V. B. Carruthers, S. D. Gilk, G. E. Ward, The Toxoplasma homolog of Plasmodium apical membrane antigen-1 (AMA-1) is a microneme protein secreted in response to elevated intracellular calcium levels. *Mol Biochem Parasitol* **111**, 15-30 (2000).
